# Alcohol, Substance Use and Psychosocial Competence of Adolescents in Selected Secondary Schools in Uganda: A Cross Sectional Survey

**DOI:** 10.9734/INDJ/2016/25387

**Published:** 2016

**Authors:** Catherine Abbo, Elialilia S. Okello, Wilson Muhwezi, Grace Akello, Emilio Ovuga

**Affiliations:** Department of Psychiatry, Nara Medical University, Japan; 1 Department of Psychiatry, College of Health sciences, Makerere University, P.O.Box 7072, Kampala, Uganda; 2 Department of Psychiatry, Faculty of Medicine, Gulu University, P.O.Box 126, Gulu, Uganda

**Keywords:** Substance use, young people, psychosocial competence, Uganda

## Abstract

**Aims:**

1) To determine the nature and extent of alcohol and substance use and 2) To describe the relationship between alcohol use and psychosocial competence among secondary school youths in Northern and Central Uganda.

**Study Design:**

This was a cross-sectional study.

**Place and Duration of study:**

Departments of Mental Health, Gulu University (Northern Uganda) and Department of Psychiatry, Makerere University College of Health Sciences (Central Uganda) between September 2011 and April 2012.

**Methodology:**

Four (4) and eight (8) secondary schools located in the rural and urban areas of Gulu and Kampala districts respectively were randomly selected to participate in the survey. A total of 3,200 students aged 12 to 24 years were recruited by proportionate multistage sampling. Data was collected using a socio-demographic questionnaire that included questions about nature and frequency of alcohol and substance use. A pre-tested self-administered survey questionnaire with scales to measure components of psychosocial competence (PSC) was administered. Data was entered in Epidata, and exported to SPSS version 16.0 for analysis. Psychosocial competence was classified as high or low depending on the responses in the sub-scales of decision making, self efficacy, empathy, emotional awareness, coping with stress and emotions, and accurate self-assessment and self-confidence.

**Results:**

A total of 2,902 questionnaires comprising of 2,502, (86.2%) from Kampala district and 400 (13.8%)) from Gulu district were analyzed. Male to female ratio was 1:1 with an age range of 12 to 24 years and a mean of 16.5. About 70.1% had ever used alcohol and substances. Only 39.1% used substances regularly. The commonest substance used was alcohol (23.3%), followed by *kuber* (10.8%), *khat* (10.5%), aviation fuel (10.1%), cannabis (9.2%) and cigarettes (5.9%). Respondents from the Gulu district were twice more likely to use all substances. Users and regular users from the North Northern Uganda had lower psychosocial competence. Factors significantly associated with non-use of alcohol were high levels of self-confidence, non-use of cannabis and kuber and age. In the alcohol user groups, a high level of coping was associated with discontinued and experimental use of drugs of abuse.

**Conclusion:**

More than two-thirds (70.1%) of young people in this study had ever used substances of abuse only once and slightly over a third had used it regularly. From the perspectives of service provision, mental health promotion and prevention of illicit substance use, school mental health programmes that target both non-users and users are recommended.

## 1 Introduction

The use of alcohol and other substances during adolescence and early adulthood has become a serious public health problem in Uganda. The global burden of disease projected that tobacco, alcohol and illicit drugs were respectively the 2nd, 9th and 20th leading cause of mortality globally [1]. This report further projected that tobacco smoking alone would lead to 1 billion deaths globally during the 21st century [1]. The World Health Organization’s global status report on Alcohol, 2004 stated that Uganda had one of the highest alcohol and substance abuse rates in the World [2]. With over half of Uganda’s population aged below 24 years, school going adolescents and young people are part of these statistics [3]. A study done on drug and substance abuse in schools of Kampala and Wakiso found that between 60 to 71% of students used illicit drugs with alcohol and cannabis taking the biggest percentages [4].

Given the serious consequences of drug and alcohol abuse, considerable effort has been directed toward adults who have developed health problems with the low success rates [5]. In research and clinical studies, adolescent alcohol and substance use has been relatively neglected [6]. In Uganda, there is a paucity of services and treatment programs, with the few available treatment programs and models targeting adults without appropriate attention to different developmental and child protection needs. In addition, there is no policy to guide any implementation of services to control alcohol and illicit substance use among children and adolescents in Uganda.

The continued use of these substances of abuse has a spectrum of adverse outcomes including psychological, physical, social and legal problems. Among adolescents with substance use problems, co-occurring mental disorders are common and serious [7]. In general, research has shown that individuals with co-occurring disorders (also called dual diagnosis) have more severe psychiatric symptoms, are more difficult to treat, incur greater costs, and have worse overall outcomes than persons with only one disorder [7].

Physical adverse health effects have been shown in adolescent smokers, including effects on the lungs [8]. While many of these conditions, particularly the physical ones develop after a chronic use spanning many decades, and are therefore rare in children and adolescents, an understanding of substance use and substance use problems during adolescence is critical to any approach aimed at lessening harmful consequences of illicit use of addictive substances. This is because it is during childhood and adolescence that the use of addictive substances typically first occurs [6]. Some studies suggest that if illicit substance use is not initiated by age 21, it is unlikely to ever be initiated [6,9]. Furthermore, age at initiation of illicit substance use has consistently been shown to be associated with higher lifetime consumption, more risky patterns of use and, a high level of severity of dependence [10]. Studies suggest that the younger an individual is at the onset of substance use, the greater the likelihood that a substance use disorder will develop and continue into adulthood [10]. Furthermore, it is stated that more than 90% of adults with current substance use disorders started using illicit drugs of abuse before age 18; half of those began before age 15 years [11]. Thus, it is clear that early onset use is a robust indicator of risk for future substance related physical and mental health problems.

There is also a growing recognition of the high cost of treatment and of the inability of existing treatment programs to keep up with increasing demand. Half of the admissions in the Ugandan National Mental Referral Hospital are young people with alcohol and substance use disorders [12]. These observations stimulate interest in primary prevention of alcohol and other drugs of abuse in adolescents. High psychosocial competence is one of the factors that has been stated to be protective against progressing to problematic use of alcohol and other substances [13,14], and it is a critical starting point for policy reform aimed at promoting mental health, and for preventing and controlling illicit substance use by young people in Uganda.

This article focuses on the use of alcohol and other substances of abuse among young people in secondary schools in selected districts of Central and Northern Uganda. In this article, we advance the understanding that alcohol and illicit substance use occurs along a spectrum ranging from beneficial to problematic use. We also support the proposition that harmful alcohol and substance use is facilitated by poor problemsolving capacity and low psychosocial competence. This conceptualization emphasizes the public health-based understanding of substance use instead of binary categorical approach of "use" vs. "abuse" [15]. Viewed in this way, alcohol and substance use-related problems can be understood as occurring on different levels of use associated with different types of problems and levels of psychosocial competence, with young people moving along the different levels [16]. Many young people will experiment with alcohol and available substances of abuse and stop while others may go on to recreational use and a few may get addicted and develop varying types and levels of complications [16].

In this paper, we define alcohol and substance use as lying along a continuum. This may be a one-time use, regular use or problematic use. Problematic use can further be classified as i) substance abuse which involves the use of substances despite persistent social, interpersonal or other problems caused by the use of the substance [17] and ii) substance dependence which is a more severe disorder entailing signs of physical or psychological tolerance or dependence [17]. Studies have shown that there are factors that cause some adolescents to be particularly vulnerable to problematic use of alcohol and substances [18] [6]. Factors associated with resilience are termed as assets. These are positive factors that reside within the individual and they include psychosocial competence and resources. They help youth to overcome stressors and other forms of risk. There are other protective factors that are external to the individual; notably, a supportive family environment or caring relationship with at least one adult [19]. The importance of identification of these factors, and their impact upon the progression or not of substance use in particular individuals underscores the importance of prevention and early intervention programs for young people. Botvin and others studied the effectiveness of a drug abuse prevention program and enhanced life skills (psychosocial competence) training [20,21]. At 6 year follow-up, this study showed that self-reported substance abuse was 44% less in the intervention group and poly-drug abuse was 66% less [21].

Our study aimed to determine the nature and extent of alcohol and illicit substance use and to describe the relationship between alcohol and illicit substance use and psychosocial competence in young people in secondary schools in Northern and Central Uganda.

## 2 Materials and Methods

This study set out to answer the following research questions; a) what the nature and extent of alcohol and illicit substance use among young people in secondary schools in Northern and Central Uganda was?, and b) what was the relationship between alcohol and illicit substance use and psychosocial competence of young people in secondary schools in the study areas?.

### 2.1 The Context of the Study

The experience of alcohol and substance use has always been rooted in the social context. The following section describes the context of the study in relation to the two study regions.

#### 2.1.1 Gulu district in Northern Uganda

Gulu District was created in May 2011 with a total population of about 298,500, with 50% being children and adolescents less than 18 years [22]. The district headquarters at Gulu are located approximately 340 kilometers (210 miles), by road, north of Uganda’s capital city, Kampala. The main economic activity in the district is subsistence agriculture, in which over 90% of the population is engaged. For over 20 years (1986-2008), Gulu district had experienced a civil war between government forces and the Lord’s Resistance Army. During this time, more than 90% of the population was internally displaced and lived in camps clustered around towns and trading centers for their protection. Many school children were abducted since 1986; more than 100,000 people were killed as direct and indirect action of the LRA. Over 40,000 children became night commuters, leaving their villages at night and staying in towns including centers, hospitals, storefronts, verandas of Gulu Municipality [23]. Many of commuter children were abused during their nightly stay [24]. Social services such as schools and hospitals became dysfunctional. More schools were closed during the time of the war causing a high drop out of students. Schools in camps did not have the resources for teachers to do a proper job of teaching children [18]. Currently, the north is rebuilding, hospitals and private clinics have opened; schools have reopened, and those that were relocated from the rural areas to the camps have been moved back to their previous locations [25].

#### 2.1.2 Kampala district in Central Uganda

Kampala district lies within the Kingdom of Buganda, in Central Uganda. In this region, the first organized resistance was by the Bataka movement in the 1920s [26]. In 1949, discontented Baganda rioted and burnt houses of pro-government chiefs [26]. Other moments of violence in the history of Kampala include the Nakulabye massacre in 1964, the Kabaka crisis of 1966 and the Coup de tat of Idi Amin in 1971. Amin’s time was characterized by general fear and insecurity as thousands of people reportedly disappeared mostly from Kampala. This period was followed by the political unrest that characterized the power vacuum of the late 1970s and early 1980s [26]. Notably, the ousting of Id Amin and Obote in 1979 and 1985 respectively. The coming in of the current president in 1986 was not peaceful either. In the past few years notably from 2006 Kampala has experienced a number of riots that usually may start as a peaceful demonstration and turn into riots [27]. A number of people have been injured and a few lives lost during skirmishes between security forces and civilians. In addition, there were the September 2009 Buganda riots, the terrorist attacks and school fires that rocked. A significant percentage of the district is slums. According to UN-HABITAT, 44 percent of Kampala’s population lives in unplanned, underserviced slums. Informal settlements cover up to 25 percent of the city’s total area [28].

### 2.2 Selection of Schools

The regions were selected purposively eight (8) were randomly selected secondary schools in Central Uganda. (Four government and four Privately owned schools). From each category of schools, we selected Two (2) located in Urban and two (2) in Peri-urban areas. From Northern Uganda, four schools were selected: 2 governments aided and two privately owned schools. There were no private secondary schools in the rural areas of Gulu district so all the private schools were selected from the urban area.

The list of private and government schools in rural and urban locations was accessed from respective District Education Officers and Kampala City Authority. The schools were then separated regionally and then randomly selected. Although the researchers had decided to select only eight schools, a total of twelve schools were selected for Kampala District. This was done in order to have replacement already selected in case of any refusals.

### 2.3 Study Design

This was a cross sectional study.

### 2.4 Sample Size and Sampling Procedure

The sample was calculated from Kish Leslie formula for survey studies [29]. Assuming a 50% occurrence of psychosocial competence, for a 95% confidence interval and a precision of 0.05, a total of about 400 young people per school was estimated. This gave a total sample size of 3200 young people for the twelve schools. At the selection of the schools, eight schools were selected from Kampala district. The size of the sample in each stratum was taken in proportion to the size of the stratum (proportional allocation).

Sampling of students at different schools: The number of students interviewed per class was calculated from the proportion of the class multiplied by the number to be interviewed. Senior one and senior five were left out because they had not reported to schools by the time of the study.

### 2.5 Study Instruments

#### 2.5.1 Socio-demographic questionnaire

All students completed a demographic data sheet, which had questions on gender, age, class in school, religious affiliation, parenthood status, orphanhood status (for orphans), experience of domestic violence, nature of housing, number of rooms in a house where they lived and history of mental illness in the respondent and family.

#### 2.5.2 The Emotional Competence Inventory (ECI)

The ECI was used to assess psychosocial competence. The ECI is a 360-degree tool designed to assess the emotional and social competencies of individuals. The test is based on emotional competencies identified by Dr. Daniel Goleman in working with Emotional Intelligence [30]. Only the desired attributes were extracted and assessed on a Likert scale. Decision making/problem solving was assessed on 5 point Likert scale of; (1) almost always, (2) usually, (3) about half the time, (4) rarely and (5) never. Self efficacy, accurate self assessment and self confidence were assessed on 5 point Likert scale of; (1) strongly disagree, (2) disagree, (3) undecided, (4) agree and (5) strongly agree. Empathy, emotional awareness and coping with emotions were assessed on 6 point Likert scale of; (1) always, (2) very frequently, (3) occasionally, (4) rarely, (5) very rarely and (6) never. Coping with stress was assessed on 5-point Likert scale of; (1) very much, (2) often, (3) sometimes, (4) rarely and (5) not at all. The internal consistency of the scale items used was within acceptable range of cronbach’s alpha 0.60.

#### 2.5.3 Measures of substance use

Alcohol and substance use was assessed by asking a question: ‘have you ever used the following; alcohol, marijuana, khat, kuber, petrol/aviation fuel, cigarettes, others? These substances were listed in the questionnaire. This question was asked to identify lifetime users. The questions that followed asked about the frequency of taking alcohol and other substances. An example of a question asked under this genre was; how often do you drink alcohol? Students could answer by ticking off the number of times they had used any of the substances: 1= never, 2=tried but don’t use them now, 3=once a year, 4=once a month, 5= 2 to 3 times a month, 6=once a week, 7=a few times a week. This question also served as validating question [31,32]. According to the Health Behaviour in School Aged Children (HBSC) standard [33], the results on both answers were combined and recoded into five substance use subgroups: 1) those who had never used (nonuse); 2) those who tried but did not use them then (discontinued use); 3) those who used once a year (experimental use); 4) those who reported using any of the substances between once a month and 2-3 times a month (regular nonheavy use); 5) those who reported using it once a week and a few times a week (regular heavy use).

For the purposes of this article, ‘use’ is defined as any one time use of alcohol or any substances, ‘non- use’ as not ever taken alcohol or other substances; ‘regular heavy use’ defined as using any of the substances at least once a week and ‘regular non heavy use’ as using any of the substances once a month or more and excludes those who have never used any substances and experimental users.

### 2.6 Data Management, Analysis and Handling of Confounding Factors

Data was entered in EpiData Version 3 and exported to the Statistical Package for Social Scientists (SPSS) version 16.0 for cleaning, editing and analysis. We compared young people from central and northern Uganda on selected socio-demographics, using frequency distributions and the two-way contingency table analyses. In order to incorporate multistage sampling design in our survey analyses, we chose SPSS complex Samples model using robust standard errors to obtain 95% confidence intervals and p values in a weighted and multistage sample [34]. Alcohol use was included in the model as a dependent variable, dichotomized into ‘use’ (reference category) and ‘non use’. Psychosocial competence levels on each of the eight components were included as factors and confounding variables as covariates. These variables were included in a Logistic regression model.

To investigate the association between frequency of alcohol use and psychosocial competence, a five-category alcohol use variable was created (see ‘measures’ section): non-use (reference group), discontinued use, experimental use, regular non heavy use and regular heavy use. This five-category variable was included in the model as an dependent variable while correcting for age, gender, nature of housing, experience of violence orphanhood and family history of mental illness as independent variables. All analyses were carried out with SPSS version 13 for Windows. Level of significance was set at p ≤ 0.05.

## 3 Results

### 3.1 Socio-demographic Characteristics

Out of the targeted sample of 3,200, data from 2,902 (Response rate 90.7%) young people was collected using questionnaires. To be consistent with the population of targeted students in the Kampala and Gulu, proportionate sampling was used. Consequently, out of the 2,902, 2,502 (86.2%) participants came from the Central region (Kampala) while 400 (13.8%) were from the Northern region (Gulu). Male to female ratio was 1:1 with age range of 12 to 24 years and a mean age of 16.5 years. Respondents from the Northern Uganda were more likely to be males, of age group17-20 years, Christian, to be double orphaned, have family history of mental illness and more likely to experience domestic violence but less likely to come from homes living in semipermanent or permanent houses and, such homes to have 2 or more rooms (Table 1).

### 3.2 Nature and Extent of Alcohol and Substance Use

When the following direct question was asked: ‘have you ever taken the following: (1) *alcohol,* (2) *marijuana,* (3) *khat,* (4) *kuber,* (5) *petrol/aviation fuel, (6) cigarettes,* (7) *any other substances?* 36.3% of the respondents reported that they had ever used the above substances. Of these, 66% were from Northern region. The commonest substances that had ever been used were; alcohol (19.3%) followed by kuber (4.4%), cigarettes (3.9%), marijuana (2.9%), aviation fuel (1.9%), and *khat* (1.7%). Other substances mentioned included cocaine and heroin (2.2%). When a validating question of ‘*how often (if ever) do you drink alcohol beverages, smoke marijuana etc*’ was asked; 70.1% had ever used alcohol and substances. The commonest substance was alcohol (23.3%) followed by *kuber* (10.8%), *khat* (10.5%), aviation fuel (10.1%), cannabis (9.2%) and cigarettes (5.9%). Respondents from the North were twice more likely to use all substances than those from Central Uganda (Table 2). Among the users, again respondents from the North were more likely to be regular heavy users (defined as taking any substance at least once a week) of alcohol, marijuana, aviation fuel and cigarettes. The differences between regular heavy users and regular non heavy users in regard to region were however not statistically significant (See Table 2).

### 3.3 Alcohol and Psychosocial Competence

Non-users of alcohol in the central region had higher percentages of a low score on six (6) of the eight components of PSC. They were more likely to have low levels of PSC on the subscale of empathy (*P*=.01), emotional awareness (*P*=.04) and coping with emotions (*P*=.001). Nonusers from Northern Uganda had higher percentages of low score of PSC on four (4) of the components i.e empathy, emotional awareness, coping with emotions and stress. They were more likely to have lower percentages of low score levels of PSC of decision-making (*P*=.03) and self-confidence (*P*=.01). In the central region, regular non-heavy use of alcohol was significantly associated with coping with stress. They were less likely to have low levels of PSC on coping with stress. Generally, there was a tendency of the northern region to have higher percentages of low scores on PSC on five (5) of the eight components (Table 3), none of which reached significant levels. Non-users of alcohol in Central Uganda had low PSC competence; however users and regular heavy users in Northern Uganda had lower PSC (see Table 3).

## 3.4 Results of Multiple Logistic Regressions

To control for the multiple explanatory variables on alcohol non-use, multiple logistic regression was done. In this model, self-confidence, non use of cannabis and kuber, and age emerged as significantly associated factors of non-use of alcohol. Young people with high levels of self-confidence were more likely to be non-users of alcohol (*P*=.0001, adjusted OR =1.204, 95% CI =1.147−1.260). Non-users of cannabis and Kuber were also likely to be non-users of alcohol (*P*=.001; Adjusted OR =1.050; 95% CI=7.477−1.260) and *P*=.02; OR=2.688; 95% CI=2.007−3.601) respectively. The age group of 17-20 was less likely to be non-users (*P*=.003; Adjusted OR =0.713; 95% CI= 0.630−0.807).

## 3.5 Association between Alcohol Use and Psychosocial Competence in Different User Groups

High levels of components of psychosocial competence of self-confidence, coping with stress and emotions were associated with discontinued and experimental use respectively. Those with high levels of self-confidence were less likely to discontinue use while high levels of PSC on the component of coping with stress were more likely to have discontinued use. Those with PSC high levels on the component of coping with emotions were about 2 times more likely to be experimental users. The age group 17-20 emerged a strong predictor of the whole spectrum ranging from discontinued to regular heavy use (Table 4).

## 4 Discussion

### 4.1 Key Findings

For students aged 12 to 24 in randomly selected secondary schools in Northern and Central Uganda, 70.1% of respondents had ever used alcohol and substances. We found a discrepant level of nearly twice when a validation question was asked. This discrepancy in the rates of alcohol and substance use demonstrated by the two questions supports the view that response validity of substances use is highly dependent on the construction of the question, procedures for administration, investigators’ perceived intentions and respondents’ cognitive fitness [31,32]. Since the illicit use of substances of abuse is a criminal offence in Uganda, and their use often attract societal disapproval among relatives, friends and family, a much lower rate of positive answers would be expected, thus necessitating the use of a validation question. This finding is further supported by a study done in adults in IDP camps in Northern Uganda by Roberts and others in 2008 which revealed very low rates of alcohol and outright denial of alcohol use by interviewees who were drunk even at the time of interview [35].

Only 39.1% of our respondents used substances regularly. The commonest substance was alcohol 23.3%, Kuber 10.8%, Khat 10.5%, Aviation fuel 10.1%, Cannabis 9.2% and cigarettes 5.9%. The finding that alcohol is the substance most commonly used by secondary school youth is consistent with previous studies conducted among secondary school youth [3,4,36–38]. The somewhat new finding here is that *Kuber* being the second most common illicit drug used. Not much is known about this drug that is thought to have originated from India and is being sold in Ugandan supermarkets in sachets similar to tea bags disguised as mouth freshener since 2009 [39]. It is thought to be a CNS stimulant, libido enhancing, highly addictive with some of its users experiencing psychotic and or depressive like symptoms [40].

When considering the continuum of alcohol use by gender, males in this study generally had higher prevalence rates of discontinued, experimental, regular non-heavy and regular heavy use than females. Respondents in Northern Uganda were twice more likely to use all substances. The risk of having low levels of psychosocial competence among respondents from the Central was high among non-users of alcohol and other substances. While both users and regular-heavy users from Northern Uganda had lower levels of psychosocial competence. This finding may mean that use and non-use of alcohol and substances in the two regions may be influenced by same factors differently. One explanation for this finding may be that resilience may be content and context specific, i.e. a young person may be able to overcome one type of risk but unable to overcome other types of risks. Researchers have found that different assets may be associated with different risk and outcome pairings, as in our study, this makes it difficult to identify universal protective or risk factors [41].

Holding the region constant, in multiple logistic regression, factors found to be significantly associated with non use of alcohol are self esteem, use of cannabis, *kuber* and age. Young people with high levels of self-esteem were more likely to be non-users of alcohol. And among the users, those with high levels of self-esteem were less likely to have discontinued use. Self esteem is about how we rate or appraise ourselves and this attribute is closely related to self-confidence, a measure of one’s beliefs about one’s own judgment, skills and abilities. The two concepts sometimes are used interchangeably. This finding may seem contradictory but it is not far from what is in the literature. Previous studies have not provided conclusive evidence about the relationship between self esteem and alcohol use or non use [21,42]. For instance, despite the theory positing a negative relation between self-esteem and alcohol use, empirical findings have indicated that in certain situations, delinquent activities (e.g., alcohol use) can enhance self-esteem [43,44]. Some of the explanations for this finding may be that rapid developmental change occurs during adolescence and thus a lack of stability in either alcohol use or self-esteem could influence the statistical reliability of their relations with one another [35]. Further, the operative mechanisms that link self-esteem and alcohol use are likely to be complicated, and thus are not necessarily straightforward so as to delineate adequately [21,42].

In this study, the finding that non users of cannabis and Kuber were also likely to be non users of alcohol was not surprising as previous studies have indicated that most 13 and 15 year olds in Scotland in 2002 were not regular users of any substance (66%) [45]. The age group of 17-20 was less likely to be non-users but more likely to be have discontinued, experimented, and was regular non-heavy and regular and heavy use of alcohol and illicit drug use. By Ugandan laws, 18 years is the age of the majority and so parental supervision drastically reduces. This then is a key period for the Ugandan adolescent in this age group for experimentation with a wide range of behaviors and lifestyle patterns including experimenting with alcohol without quilt. From a developmental perspective, this drive to experiment with alcohol and substances may be linked to psychosocial developmental tasks [46]. Trying out new and different behaviors may be understood as part of a natural process of becoming independent and autonomous [46].

### 4.2 Use of Alcohol and Coping

Those with high PSC levels of coping with stress and emotions were about twice more likely to have discontinued or be experimental users respectively. Studies in animals have indicated that stress increases alcohol consumption and that individual animals may differ in the amount of alcohol they consume in response to stress [47]. Prolonged stress in infancy may permanently alter the hormonal stress response and subsequent reactions to new stressors, including alcohol consumption [48,49]. Sigvardsson and others reported an association between certain types of alcoholism and adverse early childhood experiences [50]. Part of our sample was drawn from the northern region that has experienced war for a long time period that may correspond with time of birth of most of the respondents.

### 4.3 Limitations of the Study

Although we made all efforts to counter some of the potential limitations of this study, there are some aspects of the research that may limit the interpretation of our findings. One is the reliance on self-report questionnaire and data. Responses to sensitive questions about undesirable or illegal behavior may be biased and subjective. However, having prior knowledge that response validity of illicit substance use is highly dependent on the construction of the question, procedures for administration, investigators’ perceived intentions and respondents’ cognitive fitness helped us in preparing beforehand. The administration of the questionnaires in school classes, assuring anonymity, making clear our intentions and asking a validating question as was done in this study, might have helped to generate somewhat reliable and valid data [31,32]. A limitation of conducting a school survey is that adolescents may be absent from school as a result of alcohol and substance use and the same adolescents may also possibly have low levels of psychosocial competence and poor coping mechanisms. This bias could have probably resulted in an underestimation of the strength of the association between alcohol use and psychosocial competence. However, during enrollment, the absent students were replaced with the next available students on the day of questionnaire administration. In addition, the timing of the study- at the beginning of term, when academic stress may be less may have minimized this bias. The study age group of 12 to 24 is a period of rapid developmental change. We used regression models that may reflect static views of development. In our analysis, the ages were grouped into 12-16, 17-20 and 21-24 to correspond with early adolescence, mid adolescence and late adolescence respectively. Despite this age grouping, our results may not represent the best approach to capturing possible dynamic relations between psychosocial competence and alcohol use. In light of the possible dynamics underlying psychosocial competence and alcohol use in young people, it is important that models be developed that can account for change reliably as part of the developmental mechanisms linking psychosocial competence with alcohol use [42]. Finally, because of the cross-sectional design of this study we cannot therefore make inferences on causal relations.

## 5 Conclusions

In this study, about three quarters of young people had ever used substances only once and slightly over a third use it regularly. Of the substances evaluated, alcohol is the commonest, followed by kuber while cigarettes are the least used. Factors found to be significantly associated with non-use of alcohol are high levels of self-confidence, non-use of cannabis and kuber, and age group of 17-20 years. In the alcohol user groups, a high level of coping was associated with discontinued and experimental use.

Young people who have difficulties adjusting to emotional and life difficulties try to escape from their problems by using alcohol or illicit drugs [51]. With time, the amount of life difficulties they have to cope with exceeds their ability to respond resulting in the inability to achieve desired goals [52]. This overload is experienced at school, families and social lives. It is therefore necessary that efforts are directed at promotion of psychosocial competence e.g. problem solving skills; device strategies to strengthen self-confidence; strategies to cope with stress, anxiety and depression. Further, setting up school mental health program to promote mental health, identify and treat mental health problems early and lastly, support to families of vulnerable young persons including the identification of family members with mental health problems [52,53].

## Consent

Assent and or consent was sought from all study participants at the time of recruitment. Participants below the age of 18 years took detailed consent forms in English and local language to their parents or guardians. The signed forms were brought back to the research assistants on the day of administration of the questionnaire. All those who declined to participate in the study were treated with respect and without prejudice. What to expect as a participant was made clear to all respondents. Confidentiality of information supplied by research participants and the anonymity of respondents were given utmost respect. All authors hereby declare that all researches have been examined and approved by the appropriate ethics committee and have therefore been performed in accordance with the ethical standards laid down in the 1964 declaration of Helsinki [54] and in accordance with the Uganda’s National Guidelines on the responsible conduct of research involving human research participants.

## Ethical Approval

Ethical clearances were obtained from the Research and Ethics Committees of Makerere University Medical School (Uganda) Reference number- REC REF 2011-232 and Uganda National Council for Science and Technology Committee on study of Human Subjects Reference number HS 1099. Administrative clearance was obtained from Ministry of Education and Sports as well as relevant District Education Officers. The head teachers of the sampled secondary schools allowed the study in their schools.

## Figures and Tables

**Table 1:** Socio-demographic characteristics of the study participants in Central and Northern region


Variables	Region			
	Central N (%)	Northern N (%)	*P* value	OR (95%CI)

Age				
12-16	1396 (55.8)	181 (45.3)	Ref	Ref
17-20	1052 (42.0)	215 (53.8)	<0.000	1.57 (1.27-1.96)
21-24	55 (2.2)	4 (1.0)	.57	0.40 (0.17-1.67)
Gender				
Male	1228 (49.1)	231 (57.8)	.001	0.71 (0.57-0.87)
Female	1274 (43.9)	169 (42.3)		
Class				
S2	785 (31.4)	122 (30.5)	Ref	Ref
S3	705 (28.2)	105(26.3)	.78	0.99 (0.72-1.28)
S4	584 (23.3)	112 (28.0)	.15	1.23 (0.93-1.65)
S6	428(16.7)	61(15.1)	.41	0.62 (0.19-2.31)
Religion				
SDA	157 (6.3)	3 (0.8)	Ref	Ref
Protestant	583(23,3)	131(32.8)	<0.000	11.76 (3.57-46.84)
Catholic	744 (29.7)	219 (54.8)	<0.000	15.41 (4.71-60.98)
Muslim	580 (23.2)	17 (4.3)	.78	1.53 (0.42-6.67)
Pentecostal	404 (16.1)	30 (7.5)	.02	3.89 (1.11-16.21)
Others (JW&Trad)	34 (1.4)	0.0	1.00	0.00 (0.00-10.86)
Both Parents alive				
Yes	1774 (70.9)	224 (56.0)	<0.000	1.90 (1.53-2.35)
No	728 (29.1)	176 (44.0)		
Orphanhood				
Maternal/Paternal Orphan	560 (29.1))	50(13.1)	<0.000	8.40 (5.71-12.38)
Double Orphan	168 (7.2)	126(30.9)		
Domestic Violence				
Don’t want to say	111(4.4)	19 (4.7)	Ref)	Ref
Yes	585 (23.0)	139(34.8)	<0.000	0.005 (0.004-0.007
No	1806(72.6)	248 (60.5)	<0.000	0.003 (0.002-0.004))
Nature of Housing				
Hut	114 (4.6)	205 (51.3)	Ref	Ref
Semi permanent	683 (27.3)	99 (24.8)	,0.000	0.08 (0.06-0.11)
Permanent House	1705 (68.1)	96 (24.0)	<0.000	0.03 (0.02-0.04)
Number of rooms				
1 room	351(13.7)	142(35.1)	Ref	ref
2 rooms	692(23.2)	89(22.0)	<0.000	0.29 (0.21-0.39)
3 rooms	625(24.5)	93(23.0)	<0.000	0.33 (0.24-0.45)
More than 3 rooms	987(38.6)	80(19.8)	<0.000	0.21 (0.16-0.28)
Family history of mental illness				
Yes	628 (25.0)	137(34.3)		
No	1874 (74.9)	263(65.8)	<0.000	0.64 (0.51-0.81)


**Table 2:** Use and non-use of substance by region (Central and Northern)


Substance of use	Region			
	Central *(N %)*	Northern *(N %)*	*P*-value	Crude ORs (95% CI)

**Alcohol**				
Use	516 (20.6)	159 (39.8)**		
Non use	1986 (79.4)	241 (60.3)	**<0.000**	0.39 (0.32-0.49)
**Marijuana**				
Use	201 (8.0)	67 (16.8)**		
Non use	2301 (92.0)	333 (83.3)	**<0.000**	0.51(0.38-0.68)
**Khat**				
Use	243 (9.7)	70 (17.5)**		
Non use	2259 (90.3)	330 (82.5)	**<0.000**	0.51(0.38-0.68)
**Kuber**				
Use	247 (9.9)	59 (14.8)**		
Non use	2255 (90.1)	341 (85.3)	**0.003**	0.63 (0.47-0.86)
**Fuel**				
Use	239 (9.6)	55 (13.8)**		
Non use	2263 (90.4)	345 (86.3)	**0.01**	0.66 (0.48-0.91)
**Cigarettes**				
Use	127 (5.1)	45 (11.3)**		
Non use	2375 (94.9)	355 (88.8)	**<0.000**	0.42 (0.29-0.60)


*Significant at p≤0.05*

* *Use defined as any one-time use of alcohol or any substances, non-use as not ever taken alcohol or other substances*

**Table 3:** Alcohol use and non use by components of PSC and region


Components of PSC	Region							
	Central				Northern			

	Non use (N=1986) *n(%)*	Use (N=516) *n(%)*	*P*-value	Crude ORs (95%CI)	Non use (N=241) *n(%)*	*Use (N=159) n(%)*	*P*-value	Crude ORs (95%CI)

**Decision making**								
High	1513(76.2)	408(79.1)			192(79.7)	112(70.4)		
Low	473(23.8)	108(20.9)	.17	1.18(0.93-1.50)	49**(20.3)**	47(29.6)	.03***	0.61(0.38-0.97)
**Self efficacy**								
High	1461(73.6)	389(75.4)			156(64.7)	99(62.3)		
Low	525(26.4)	127(24.6)	.40	1.10(0.88-1.37)	85(35.3)	60(37.7)	.62	0.89(0.59-1.36)
**Empathy**								
High	1034(52.1)	302(58.5)			146(60.6)	99(62.3)		
Low	952(47.9)	214(41.5)	.01	1.23(1.07-1.58)	95(39.5)	60(37.7)	.74	1.07(0.71-1.62)
**Emotional awareness**								
High	1244(62.6)	349(67.6)			167(69.3)	111(69.8)		
Low	742(37.4)	167(32.4)	.04	1.25(1.02-1.53)	74(30.7)	48(30.2)	.91	1.03(0.66-1.58)
**Coping with emotions**								
High	1061(53.4)	317(61.4)			414(58.5)	96(60.4)		
Low	925(46.5)	199(38.6)	.001	1.39(1.14-1.69)	100(41.5)	63(39.6)	.71	1.08(0.72-1.63)
**Coping with stress**								
High	626(31.5)	182(35.3)			89(36.9)	59(37.1)		
Low	1360(68.5)	334(64.7)	.11	1.18(0.96-1.45)	152(63.1)	100(62.9)	.97	1.01(0.66-1.52)
**Accurate self assessment**								
High	1570(79.1)	394(76.4)			155(64.9)	93(58.5)		
Low	416(20.9)	122(23.6)	.18	0.86(0.68-1.08)	84(35.1)	66(41.5)	.24	0.78(0.52-1.18)
**Self confidence**								
High	1563(78.3)	395(76.6)			178(73.9)	99(62.3)		
Low	423(21.3)	121(23.4)	.29	0.88(0.70-1.11)	63(26.1)	60(37.7)	.01***	0.58(0.38-0.89)


**Table 4:** Association between alcohol use and psychosocial competence in different user groups


Alcohol user groups	P	Adjusted OR	95% CI

Discontinued use			
Age (17-20)	.02	1.31	1.04-1.64
Self confidence (high)	.03	0.72	0.53-0.96
Coping with stress (high)	.05	1.29	1.01-1.66
Experimental use			
Age (17-20)	<0.0001	2.34	1.49-3.67
Coping with emotions (high)	.01	2.22	1.25-3.95
Regular non heavy use			
Age (17-20)	<.0001	1.88	1.45-2.45
Regular heavy use			
Age (17-20)	<.0001	2.13	1.43-3.15


## Abbreviations

PSC: Psychosocial competence
WHO: World Health Organization
